# Supplementary material for: Modeling in higher dimensions to improve diagnostic testing accuracy: Theory and examples for multiplex saliva-based SARS-CoV-2 antibody assays
Source: PLoS One. 2023 Mar 13;18(3):e0280823. doi: 10.1371/journal.pone.0280823 (PMC10010503; doi:10.1371/journal.pone.0280823)
Supplement: S1 Appendix — (PDF) [file pone.0280823.s001.pdf]

Modeling in higher dimensions to improve diagnostic testing accuracy: theory and examples for multiplex saliva-based SARS-CoV-2 antibody assays

Rayanne A. Luke[1,2*], Anthony J. Kearsley[2], Nora Pisanic[3], Yukari C. Manabe[4], David L. Thomas[4], Christopher D. Heaney[3,5,6‡], Paul N. Patrone[2‡]

1 Department of Applied Mathematics and Statistics, Johns Hopkins University, Baltimore, MD, USA
2 Applied and Computational Mathematics Division, National Institute of Standards and Technology, Gaithersburg, MD, USA
3 Department of Environmental Health and Engineering, Johns Hopkins University, Baltimore, MD, USA
4 Department of Medicine, Johns Hopkins University, Baltimore, MD, USA
5 Department of Internal Health, Johns Hopkins University, Baltimore, MD, USA
6 Department of Epidemiology, Johns Hopkins University, Baltimore, MD, USA

*Corresponding author. E-mail: rluke3@jhu.edu
‡Senior authors. Questions about assay design can be directed to Christopher D. Heaney at cheaney1@jhu.edu.

S1 Appendix

Details of models

Our mathematical models are probability distributions that describe the chances of observing a positive or negative sample in 3D measurement space. To create a probability distribution, we select a parameterized model that qualitatively describes the shape of the population. Examples of distributions commonly used to model biological phenomena include the normal, uniform, beta, and exponential.

To motivate a specific choice, first note that much of the negative data have N and RBD values that are roughly proportional. Mathematically, this means that the data lies along the diagonal line $y = x$, suggesting the change of variables

$$u = \frac{x+y}{\sqrt{2}}, \quad w = \frac{x-y}{\sqrt{2}}, \quad v = z. \tag{A1}$$

Additionally, the negative data fans out away from the origin along the diagonal (see Fig. 1a). This suggests that the variance of the difference w between N and RBD increases with their sum, i.e., the variable u. We empirically choose the variance σ_w of this difference to be

$$\sigma_w = \alpha \exp\left[\frac{u-\mu_u}{\beta}\right], \tag{A2}$$

for constants α and β, where μ_u is a characteristic total SARS-CoV-2 antibody level, which is yet to be determined.

A 3D distribution in variables u, w, and v was created for the negative population. We select a hybrid triple normal distribution having the form

$$N(\boldsymbol{r}) = \frac{1}{(2\pi)^{3/2}\sigma_u\sigma_w\sigma_v} \exp\left\{-\frac{1}{2}\left[\left(\frac{u-\mu_u}{\sigma_u}\right)^2 + \left(\frac{w-\mu_w}{\sigma_w}\right)^2 + \left(\frac{v-\mu_v}{\sigma_v}\right)^2\right]\right\}, \tag{A3}$$

where σ_u and σ_v are constants and σ_w is defined by Eq. A2. We use maximum likelihood estimation (MLE) to identify the model parameters $\mu_u, \mu_w, \mu_v, \sigma_u, \sigma_v, \alpha$, and β that maximize the probability of observing the negative training data [1].

Similarly, we introduce changes of variables and model the positive population. The structure of the positive data motivates a change to a spherical coordinate system:

$$\zeta = \sqrt{x^2+y^2+z^2}, \quad \omega = \arctan\left(\frac{\sqrt{x^2+y^2}}{z}\right), \quad \phi = \arctan\left(\frac{y}{x}\right), x > 0. \tag{A4}$$

This reflects the fact that the data moves out radially in 3D from the origin. We use a weighted hybrid triple normal distribution:

$$P(\boldsymbol{r}) = \frac{1}{4\pi^{3/2}\sigma_\zeta\sigma_\omega\sigma_\phi} \exp\left\{-\frac{1}{2}\left[\left(\frac{\zeta-\mu_\zeta}{\sigma_\zeta}\right)^2 + \left(\frac{\omega-\mu_\omega}{\sigma_\omega}\right)^2 + \frac{1}{2}\left(\frac{\phi-\mu_\phi}{\sigma_\phi}\right)^2\right]\right\}, \tag{A5}$$

where $\sigma_\zeta, \sigma_\omega$ and σ_ϕ are constants. MLE is used to determine the optimal model parameters using the positive training data.

Using either a known prevalence q or estimated prevalence $\hat{q}$ (see the next section), we then classify a measurement $\boldsymbol{r}$ as positive if

$$(1-q)N(\boldsymbol{r}) < qP(\boldsymbol{r}) \tag{A6}$$

and negative if

$$qP(\boldsymbol{r}) < (1-q)N(\boldsymbol{r}). \tag{A7}$$

Prevalence estimation

We can estimate the disease prevalence if it is unknown following [2]. The strategy requires dividing the measurement space into two arbitrary regions. Many clustering algorithms to select these regions exist [3, 4]; our implementation chooses these regions based on a k-means clustering of the data. For a binary classification, we use $k = 2$ clusters and assign each point in our 3D measurement space to the cluster with the closest mean. We select a region D on one side of the boundary separating the clusters and count the total number of points irrespective of their (unknown) class; call this number Q_D. We compute the integrals

$$P_D = \int_D P(\boldsymbol{r})d\boldsymbol{r}, \quad N_D = \int_D N(\boldsymbol{r})d\boldsymbol{r}, \tag{A8}$$

which are the probabilities that a known positive or known negative sample falls in the domain D. Then our prevalence estimate is computed as

$$\hat{q} = \frac{Q_D - N_D}{P_D - N_D}. \tag{A9}$$

This estimate $\hat{q}$ can be used in place of the true prevalence when q is unknown. Our estimate is unbiased. Moreover, as the number of samples increases, $\hat{q}$ will converge to q.

Local accuracy

The local accuracy, Z, of a test sample with measurement $\boldsymbol{r}$ is given by

$$Z_P(\boldsymbol{r}) = \frac{qP(\boldsymbol{r})}{qP(\boldsymbol{r}) + (1-q)N(\boldsymbol{r})} \tag{A10}$$

if the sample falls in the optimal positive domain, and by

$$Z_N(\boldsymbol{r}) = \frac{(1-q)N(\boldsymbol{r})}{qP(\boldsymbol{r}) + (1-q)N(\boldsymbol{r})} \tag{A11}$$

if the sample falls in the optimal negative domain. The denominators in Eqs. A10 and A11 give the probability that a test sample has a measurement value $\boldsymbol{r}$. See [1] for details.

References

1. Rasmussen CE, Williams CK. Gaussian processes for machine learning. vol. 2. MIT press Cambridge, MA; 2006.

2. Patrone PN, Kearsley AJ. Classification under uncertainty: data analysis for diagnostic antibody testing. Math Med Biol. 2021;38(3):396–416.

3. Abbas OA. Comparisons between data clustering algorithms. Int Arab J Inf Technol. 2008;5(3).

4. Jain AK. Data clustering: 50 years beyond K-means. Pattern Recognit Lett. 2010;31(8):651–666.
