# Supplementary figures and images for: Modeling in higher dimensions to improve diagnostic testing accuracy: Theory and examples for multiplex saliva-based SARS-CoV-2 antibody assays
Source: PLoS One. 2023 Mar 13;18(3):e0280823. doi: 10.1371/journal.pone.0280823 (PMC10010503; doi:10.1371/journal.pone.0280823)

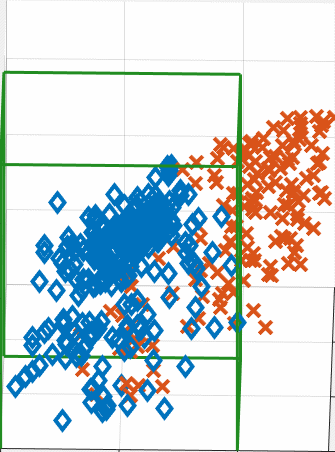

Supplement: S1 Fig — Positive samples are indicated with red Xs and negatives with blue ♦s. The green box gives the negative sample mean plus 3σ confidence interval. (GIF) [file pone.0280823.s002.gif]

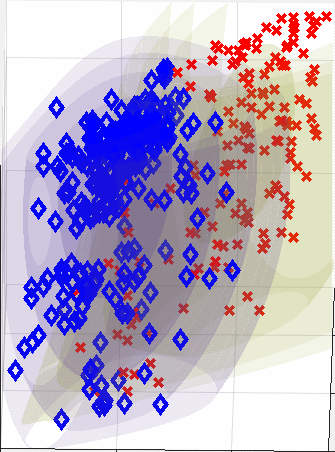

Supplement: S2 Fig — 3D probability models plotted along with the training data. Positive samples are indicated with red Xs and negatives with blue ♦s. (GIF) [file pone.0280823.s003.gif]

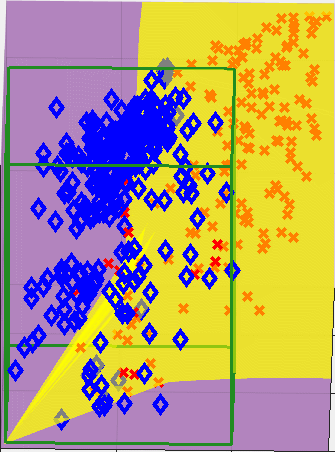

Supplement: S3 Fig — Optimal classification domains for the training data. Positive samples are indicated by red Xs and negatives with blue ♦s. The green box gives the negative sample mean plus 3σ confidence interval. (GIF) [file pone.0280823.s004.gif]

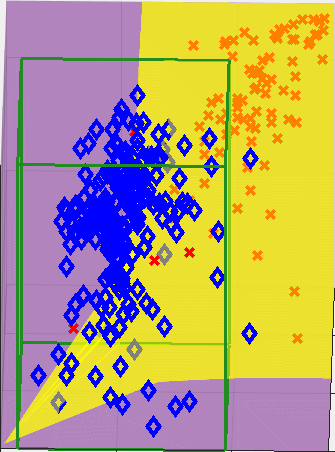

Supplement: S4 Fig — Optimal classification domains for the test data. Positive samples are indicated by red Xs and negatives with blue ♦s. The green box gives the negative sample mean plus 3σ confidence interval. (GIF) [file pone.0280823.s005.gif]

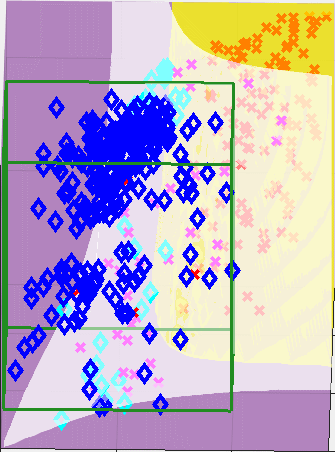

Supplement: S5 Fig — Optimal classification domains for the training data without the holdout region (white; magenta and cyan markers are indeterminate samples). Positive samples are indicated by red Xs and negatives with blue ♦s. The green box gives the negative sample mean plus 3σ confidence interval. (GIF) [file pone.0280823.s006.gif]

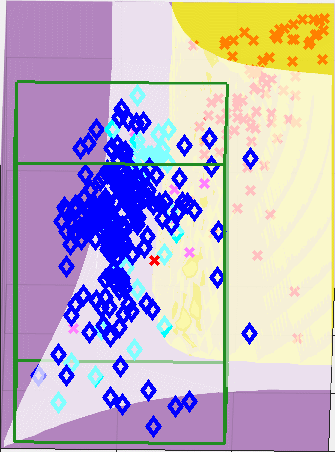

Supplement: S6 Fig — Optimal classification domains for the test data without the holdout region (white; magenta and cyan markers are indeterminate samples). Positive samples are indicated by red Xs and negatives with blue ♦s. The green box gives the negative sample mean plus 3σ confidence interval. (GIF) [file pone.0280823.s007.gif]
